# Pyrethroid Pesticide Metabolite in Urine and Microelements in Hair of Children Affected by Autism Spectrum Disorders: A Preliminary Investigation

**DOI:** 10.3390/ijerph13040388

**Published:** 2016-03-30

**Authors:** Valentina F. Domingues, Cinzia Nasuti, Marco Piangerelli, Luísa Correia-Sá, Alessandro Ghezzo, Marina Marini, Provvidenza M. Abruzzo, Paola Visconti, Marcello Giustozzi, Gerardo Rossi, Rosita Gabbianelli

**Affiliations:** 1 REQUIMTE/LAQV, Instituto Superior de Engenharia do Porto, Instituto Politécnico do Porto, 4200-072 Porto, Portugal; vfd@isep.ipp.pt (V.F.D.); mariacs31@gmail.com (L.C.-S.); 2 School of Pharmacy, University of Camerino, 62032 Camerino, Italy; cinzia.nasuti@unicam.it; 3 Computer Science Division, School of Science and Technology, University of Camerino, 62032 Camerino, Italy; marco.piangerelli@unicam.it; 4 DIMES, School of Medicine, Università di Bologna, 40126 Bologna, Italy and Don Carlo Gnocchi Foundation ONLUS, 20162 Milan, Italy; alessandro.ghezzo2@unibo.it (A.G.); marina.marini@unibo.it (M.M.); provvidenza.abruzzo2@unibo.it (P.M.A.); 5 IRCCS Institute of Neurological Sciences-Bologna, 40126 Bologna, Italy; paola.visconti@ausl.bologna.it; 6 Laboratorio NovEra srl, 62012 Civitanova Marche, Italy; LaboratorioNovEra@Pec.it (M.G.); dr.gerardorossi@libero.it (G.R.)

**Keywords:** Autism Spectrum Disorder, urine metabolites, 3-phenoxybenzoic acid, hair metals and microelements

## Abstract

The number of children affected by Autism Spectrum Disorders (ASD) is dramatically increasing as well as the studies aimed at understanding the risk factors associated with the development of ASD. Since the etiology of ASD is partly genetic and partly environmental, factors (*i.e.*, heavy metals, pesticides) as well as lifestyle seem to have a key role in the development of the disease. ASD and Control (CTR) children, aged 5–12 years, were compared. Gas chromatography coupled with trap mass detector was used to measure the level of 3-PBA, the main pyrethroid metabolite in urine in a group of ASD patients, while optical emission spectrometry analysis was employed to estimate the level of metals and microelements in hair in a different group of ASD children. The presence of 3-PBA in urine seems to be independent of age in ASD children, while a positive correlation between 3-PBA and age was observed in the control group of the same age range. Urine concentration of 3-BPA in ASD children had higher values than in the control group, which were marginally significant (*p* = 0.054). Mg results were significantly decreased in ASD with respect to controls, while V, S, Zn, and Ca/Mg were marginally increased, without reaching statistical significance. Results of Principal Component (PC) analysis of metals and microelements in hair were not associated with either age or health status. In conclusion, 3-PBA in urine and Mg in hair were changed in ASD children relative to control ones.

## 1. Introduction

Autism Spectrum Disorders (ASD) are a group of neurodevelopmental disorders characterized by social and behavior impairments. The number of children affected by ASD is dramatically increasing as well as studies aimed at identifying the risk factors associated with the development of ASD. The etiology of ASD is mostly genetic, as demonstrated by the fact that it is highly heritable; however environmental factors (*i.e.*, heavy metals, pesticides), as well as the lifestyle seem to have a key role in the development of the disease.

Hair has been proposed as a useful biological matrix to assess environmental exposure to toxic metal concentrations and to evaluate nutritional status in animal and human studies [1]. Hair has more advantages compared to other biological samples (*i.e.*, blood and urine) because it can be more easily collected. Besides, the concentration of microelements and heavy metals can be 50–100 times higher than in other matrices, and hair can give information about short and long term exposure better than plasma where biomarkers are influenced by homeostatic control systems [1,2]. Previous studies show that hair can be usefully employed to screen metal and microelement imbalance in several diseases [1,2,3,4,5,6,7,8,9,10,11,12,13], and it is a suitable biomarker if properly collected and processed [1,2,3,4,5,6,7,8,9,10,11,12,13]. However the external contaminants and hair treatments can represent a limitation for this analysis.

Toxic metal exposure has been significantly associated with ASD [4]. A decrease in Mg and Zn, together with an increase of Al, Cd, and Pb were observed in the hair of autistic children compared to control group [5]. Zn deficiency, together with an excess of Cu, were measured in very young children with ASD, where a positive correlation between Cu/Zn and the severity of ASD was observed [6]. Similarly, a deficit of Zn, Se, Ca, I, Mg, Mn, Mb, together with high levels of Al, As, Hg, Sb, Ni, Z, Pb, and V, was observed by Blauroch-Busch *et al.* in ASD children [7]. Mg and Cu were decreased in the hair of an autistic girl, while decreased Fe and increased Se levels in hair from ASD children were found compared to controls [8]. Li, I, and P were decreased in hair from autistic children compared to controls [9]. Hg, Pb, and U were higher in hair of ASD boys than in controls [10]. Similarly, increase of Cu, Pb and Hg together with low levels of Mg and Se were measured in ASD children [11]. Low Pb, As, Cd, and Hg levels were detected in ASD children compared to controls [12]. According to Farida *et al.*, the high levels of Hg, Pb, and Al measured in the hair of ASD children could be related to a diet rich in fish, environmental exposure to gasoline and use of aluminum pans by mothers, respectively [13]. From these studies, a multiple scenario on toxic metals and microelement content in hair of ASD children emerges.

In addition to metals and microelement imbalance, environmental pesticide exposure has been described as a risk factor in ASD [14,15,16,17]. A Childhood Autism Risks from Genetics and Environment (CHARGE) study underlined that organophosphate, carbamate and pyrethroid pesticides can lead to ASD, and that the critical window of exposure is during the pre-and-early-post-natal periods of life [18]. Among pesticides, pyrethroids are widely used in agriculture and homes for pest control purposes. FAOSTAT report on Italian pyrethroids market shows a substantial increase of pyrethroid use, which has increased from 47 to 164 tons in the period 1998–2013 [19]. Hence, we observed a progressive increase of the use of these pesticides in the last five years. Consumption of fresh and cooked fruit and vegetables has been linked to higher levels of exposure in the population. Furthermore, additional exposure via ingestion of contaminated household dust may occur after the indoor application of pesticides. Floor dust is one of the major sources of exposure (through hand-to-mouth contact) in infants and toddlers, contributing substantially to intake doses [20]. Pet shampoos, medication used for treating scabies, and topical lice treatments also contain these compounds. Dermatological uptake of pyrethroids can also occur during loading and mixing operations, treatment of pets, and via contact with contaminated work clothes or carpets and other textiles impregnated with pyrethroids for insect protection, such as battle dress uniforms, but the percentage of pyrethroid absorbed through the skin is less than that absorbed orally.

Although pyrethroids are excreted within 24 h, thus limiting the impact of body accumulation, they are characterized by pronounced lipophilicity, compared to other classes of pesticides, which makes them able to easily cross the blood-brain barrier and exert their toxic effect directly on the central nervous system [21,22]. The entity of exposure to pyrethroids can be evaluated by screening their metabolites in urine, where they are present as the final product of their catabolism.

The aim of the present study was to evaluate whether the urine concentration of the main pyrethroid metabolite, 3-phenoxybenzoic acid (3-PBA), and the concentration of metals and microelements in hair of children with ASD was different compared to control (CTR) children.

In fact, it has been pointed out that the presence of environmental toxicants and of higher-than-normal levels of heavy metals in hair of ASD children may be linked to the presence of polymorphisms in genes implicated in detoxification, thus linking genetic and environmental causes of ASD [23].

## 2. Materials and Methods

### 2.1. Materials

All reagents were of analytical grade. 3-Phenoxybenzoic-acid (3-PBA) (purity 98%), 2-phenoxybenzoic acid (2-PBA) (purity 98%), 1,1,1,3,3,3,-hexafluoroisopropanol (HFIP) (purity 99%), *N*,*N*-diisopropylcarbodiimide (DIC) (purity 99%), nitric acid (69%), and hydrogen peroxide solution (≥30%) for ultratrace analysis were all obtained from Sigma-Aldrich (Saint Louis: MO, USA).

### 2.2. Subjects

Two groups of ASD children and of healthy CTR children, in the same age range, were recruited by two Units in Italy: *i.e.*, by the Neuropsychiatric Unit of the Bellaria Hospital of Bologna and by the Laboratorio NovEra srl, Civitanova Marche, Italy. The first Unit collected urine samples in order to evaluate the level of pyrethroid pesticide exposure, whereas the second Unit measured metal and microelement levels in hair.

### 2.3. First Group of Children: Assessment of 3-PBA in Urine

A total of 40 children were recruited in this case-control study. Of these, 21 (17 males and 4 females) had a diagnosis of ASD, and 19 (15 males and 4 females) were CTR children. In the ASD group, mean age was 6.9 years (SEM = 0.509 years, median age = 6 years, range 5–12 years); in the CTR group, mean age was 7.4 years (SEM = 0.485 years, median age = 7 years, range 5–12 years).

All patients were admitted to the Child Neuropsychiatric Unit of the Bellaria Hospital of Bologna (Neurological Sciences Institute IRCCS-Bologna, Bologna), for assessment by means of a comprehensive diagnostic-neurological workup. Following this prior screening, ASD children with epilepsy or with electroencephalogram (EEG) abnormalities (8% and 40% of the total, respectively) were excluded from the study.

Any medical and neurological comorbidity was excluded by electroencephalography (recorded while waking and sleeping), cerebral magnetic resonance imaging, standard clinical and neurological examination, neurometabolic and genetic investigations. No infective or inflammatory disease was detected. Children did not take medications or food supplements.

ASD diagnosis was made according to the Diagnostic and Statistical Manual of Mental Disorders IV (DSM IV TR [24] criteria, Autism Diagnostic Observation Schedule (ADOS) [25] and Childhood Autism Rating Scale (CARS) [26]. CARS was between 31.5 and 47 (mean value = 39.265, SEM = 0.873). Developmental and cognitive levels were assessed by Psychoeducational Profile-3 (PEP-3) [27] and Leiter International Performance Scale-Revised (Leiter-R) [28]. The study was approved by the Ethical Committee of Bologna Health Authority (protocol number 13062).

Patient total CARS scores ranged from mild to severe and developmental levels varied from normal IQ to severe cognitive impairment.

Control group children were healthy children, recruited in the local community, with no sign of cognitive, learning, or psychiatric involvement, as clinically and anamnestically determined by the experienced clinician (P.V.). All control group children were attending mainstream school and had not been subjected to stressful events. Dietary habits have been assessed by a food questionnaire. All patients and controls were on a typical Mediterranean diet.

#### Urine Collection and Analysis of 3-PBA

Urine samples collected from ASD and CTR children were frozen and stored at −20 °C before the analysis.

Evaluation of urinary 3-PBA level requires hydrolysis to convert it into free 3-PBA that might be conjugated. Then the 3-PBA was extracted from the urine samples (1 mL) pre-treated with KOH by solid-phase extraction using Oasis Strata X C cartridge. Derivatization of 3-PBA and 2-PBA (the internal standard) was achieved by the addition of DIC and HFIP. In the final phase of the procedure, a liquid-liquid extraction (LLE) was made twice with 150 μL of n-hexane, followed by a vortex mixing during 10 min. Using a micropipette, 50 μL of supernatant were withdrawn (each time) and transferred in an “insert” placed in a vial.

A volume of 1 µL of upper layer was injected on a Thermo Trace-Ultra gas chromatograph coupled to an ion trap mass detector Thermo Polaris, operated in the electron impact ionization at 70 eV. The ion source temperature and the MS transfer temperature were at 250 °C. Operating in the splitless mode, the helium was used as carrier gas at a constant flow rate of 1.3 mL/min. The injector was maintained at 240 °C.

A program was developed in the SIM mode, based on the detection of selected ions for 3-PBA and 2-PBA (134, 140, 169, 195, 197, and 364).

### 2.4. Second Group of Children: Assessment of Metals and Microelements in Hair

Subjects of the second independent group referred on a voluntary basis to Laboratorio NovEra srl, Civitanova Marche, Italy. Parental consent was obtained from all participants. Clinical information was obtained from medical records supplied by parents. All subjects had been evaluated for DSM-IV-TR classification and assignment of ADOS parameters.

A total of 65 children were recruited in this case-control study. Of these, 29 (26 males and 3 females) had a diagnosis of ASD, and 36 (26 males and 10 females) were CTR children. In the ASD group, mean age was 7.3 years (SEM = 0.445 years, median age= 7.0 years, range 5–13 years); in the CTR group, mean age was 8.4 years (SEM = 0.703 years, median age = 7.5 years, range 2–15 years).

#### 2.4.1. Hair Samples

Hair was sampled from the nape of the neck with stainless steel scissors. Hair was washed with shampoo, rinsed, and dried 24-h before the collection. Hair was digested and afterwards underwent multi-elemental analysis with ICP-OES.

Hair samples were digested in a Microwave Digestion System: Anton Paar Multiwave PRO in closed Teflon bombs. Hair (0.300 g) was mineralized with 4 mL of concentrated nitric acid (69%) and 4 mL of ultrapure (for ultratrace analysis) hydrogen peroxide solution (≥30%). The reagent and digestion conditions were chosen in order to achieve complete mineralization and decomposition of solid phase into liquid phase. After digestion, the solutions were filled up to 25 mL with Ultra Pure (greater than 18.3 MΩ) water and filtered with a 0.45 μm, hydrophilic PTFE, 25 mm filter.

#### 2.4.2. Analytical Methods

The concentration of 41 elements: Ca, Mg, Na, K, Cu, Zn, Fe, P, Se, Mn, Cr, Co, Mo, Ge, S, V, Sb, Au, Li, Ni, Pt, Ag, Sr, Sn, Ti, W, Zr, Hg, Cd, Pd, Be, Al, As, U, Pd, Rh, Gd, and Tl in hair was determined by Inductively Coupled Plasma—Optical Emission Spectrometry (ICP-OES) (710 Agilent, Santa Clara, CA, USA) connected with ultrasonic nebulizer CETAC U-500AT+ (Omaha, NE, USA).

### 2.5. Statistical Analysis

To compare 3-PBA values in urine of ASD and CTR groups, normality tests were applied to all numeric variables, then a non-parametric test (Mann-Whitney) was used to compare ASD an CTR 3-PBA in urine. Parametric Pearson’s test was used to correlate age and urine 3-PBA in CTR group. Non-parametric correlation (Spearman’s rho) was used to correlate clinical features (CARS total score, age) and 3-PBA in urine in the ASD group. Statistical analysis was carried out using the program GraphPad Prism.

Hair samples collected from ASD and CTR groups were analyzed individually and results were expressed as parts per million (ppm), mean ± SEM. Statistical analysis, between the two groups (ASD and CTR) was carried out using the program Statistica 8.0 (StatSoft) (StatSoft Italy Srl, Vigonza, PD, Italy, 2007). Specifically, we used parametric (*t*-test) or non-parametric (Mann-Whitney) tests according to the normal or non-normal distribution of data. In particular, *t*-test was used for Zn and S with normal distribution, while Mann-Whitney was employed for Mg, Ca/Mg, and V. Differences were considered significant at *p* value < 0.05. No correction for multiple hypothesis testing was made, so the results should be viewed as exploratory and not conclusive.

#### Principal Components Analysis and Self-Organizing Maps

Many techniques were developed in order to have a better understanding of a highly dimensional dataset, *i.e.*, to find hidden relationships, to cluster or to reduce its dimensionality. In the present work we used the Principal Component Analysis (PCA) and Self-Organizing Maps (SOMs) in order to cluster our dataset in an unsupervised way.

PCA is a statistical procedure based on linear algebra, in particular on Singular Value Decomposition (SVD) that transforms the dataset using an orthonormal transformation from a highly correlated dataset to a linearly uncorrelated one. The new coordinates are called Principal Components (PC).

SOM is an unsupervised technique that projects high-dimensional data into a two-dimensional map; it is used for clustering and information visualization. This procedure, called “mapping”, preserves the topology of the data so that similar data items will be mapped to nearby locations on the SOM [29,30,31,32,33,34]. SOM analysis can provide a further method of data analysis capable of extracting some interesting information on the relationship among a large number of variabilities that cannot be represented by PCA analysis. A more complete explanation of SOMs and its results was provided in the Supplementary Materials. 

## 3. Results

### 3.1. 3-PBA Level in Urine of ASD and Control Children (CTR) in the First Group

**Figure 1:**
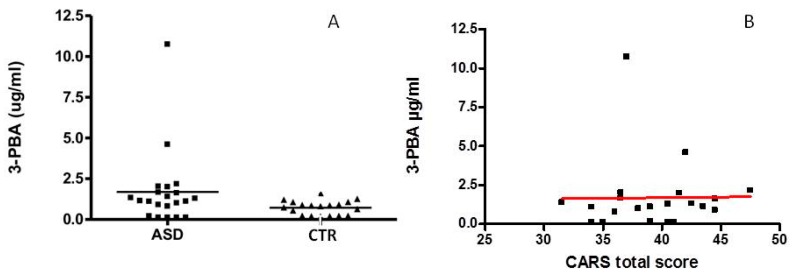
(**A**) 3-PBA level in urine in ASD and Control groups (CTR); (**B**) correlation between CARS total score and 3-PBA levels.

Figure 1A shows that the two groups differed slightly, but the difference in the urinary level of 3-PBA did not reach statistical significance (*p* = 0.054). No correlation between CARS total score and 3-PBA in urine was observed (*R*^2^ = 0.0539; *p* > 0.05). In two children (both male and six years of age) in the ASD group we found very high values of 3-PBA, reaching values from three to six times higher than the average value of the sample, respectively.

**Figure 2:**
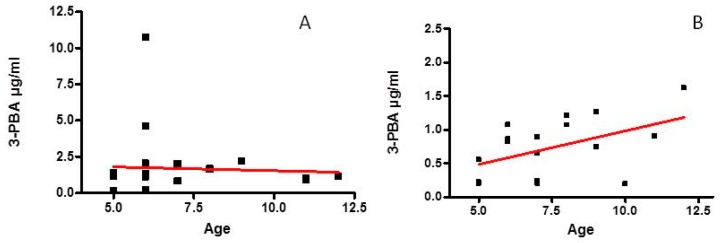
Relationship between age and of 3-PBA levels in urine of ASD (**A**) and CTR (**B**) children. Different scale was used because of different range of data.

As shown in Figure 2B, a significant increase of 3-PBA with the age in CTR children was found (*R*^2^ = 0.2196, *p* = 0.043), while no age-related change could be observed in ASD children (*R*^2^ = 0.0661, *p* > 0.05), (Figure 2A).

### 3.2. Analysis of Metals and Microelements in Hair Samples in the Second Group

Table 1 and Table 2 shows the concentration of macro/microelements and metals in the hair of ASD and CTR children. A significant decrease of Mg was found in ASD with respect to CTR children (*U* = 365, *p* = 0.0382) (Table 1). No significant difference between the groups was measured for toxic metals (Table 2). Some microelements had values close to the threshold of statistical significance: for instance, Ca/Mg ratio was higher in the ASD group (*U* = 376, *p* = 0.054), while V (*U* = 375, *p* = 0.052), S (d*f* = 63, *t* = −1.79, *p* = 0.076), and Zn (d*f* = 63, *t* = −1.71, *p* = 0.09) were lower in ASD children with respect to controls.

#### PCA Analysis

**Figure 3:**
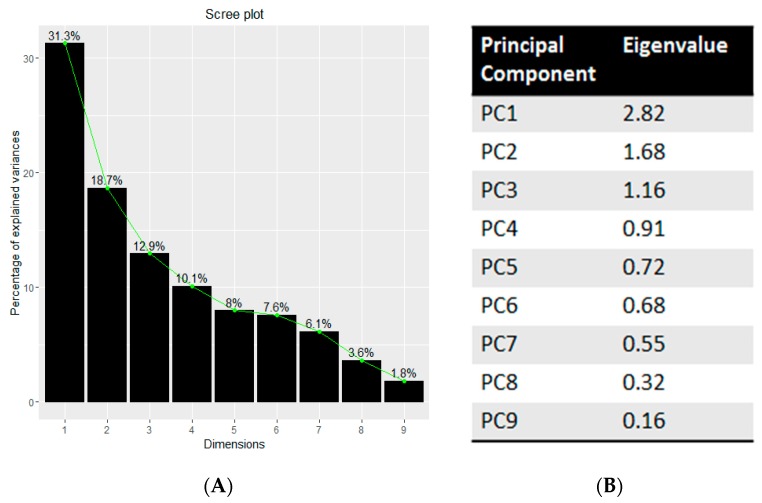
Eigenvalues (**A**) of the principal components and the relate scree plot (**B**).

PCA analysis on Mg, Cu, Zn, Hg, Pb, Fe, Se, Cd, and Al was performed using the package FactoMineR under R (Free Gnu Licence). These metals and microelements were chosen because they are changed in ASD children compared to control ones [4,5,6,7,8,9,10,11,12,13]. Figure 3A shows the eigenvalues of the principal components and Figure 3B the screen plot.

**Figure 4:**
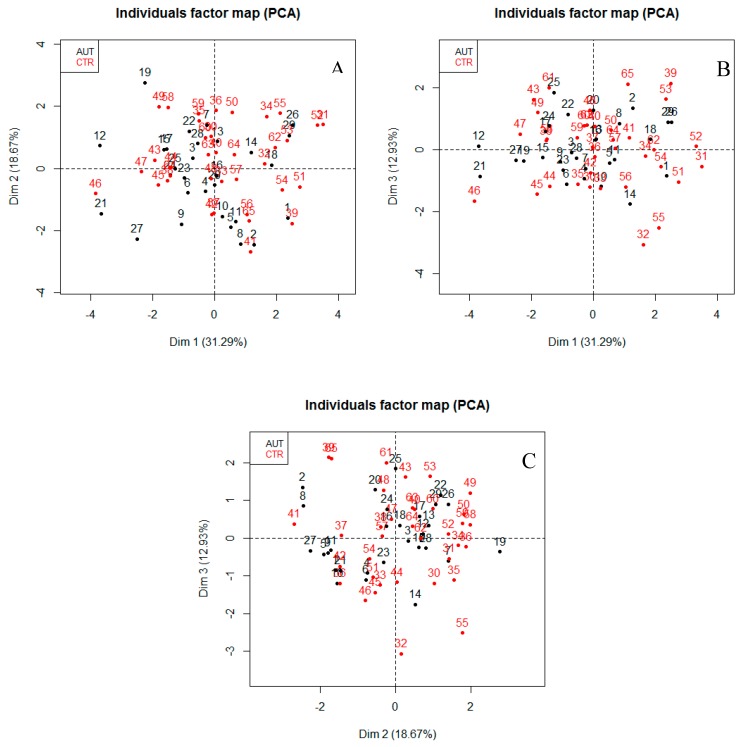
Projection onto the three projection plans PC1-PC2 (**A**); PC1-PC3 (**B**); and PC2-PC3 (**C**) of the dataset. The labels of the point are the identification numbers of the ASD children and CTR subjects.

According to the empirical Kaiser criterion, we took into account only the first three eigenvalues (which are greater than 1) and we plotted the dataset using only three principal components (PC1, PC2, PC3). In Figure 4 it is possible to observe the projection onto the three projection plans PC1-PC2 (Figure 4A), PC1-PC3 (Figure 4B), and PC2-PC3 (Figure 4C) of the dataset. It is clear that no cluster discriminating the CTR subjects from the ASD ones can be detected in the projections.

## 4. Discussion

The present data show that levels of 3-PBA, the main pyrethroid metabolite, were marginally higher (*p* = 0.054) in the urine of a group of ASD children in comparison with those of CTR children. Children included in this study did not live in rural areas and they are on a Mediterranean diet, so it could be hypothesized that exposure to pyrethroid pesticides derives from diet and residues of indoor application. It should be pointed out that one limitation of our study is the number of children included; it may be hypothesized that a larger sample may tilt the results toward a significant difference in 3-PBA urine levels between cases and controls. A significant direct relationship between 3-PBA levels and age was demonstrated in CTR children alone, whereas no correlation was found in ASD children, probably owing to the higher dispersion around the mean of the 3-PBA concentration values in ASD than in CTR children. It may be hypothesized that changes of 3-PBA concentration could be related to the longer pyrethroid exposure in children 12 years old with respect to five year olds.

Previous studies have shown that a vulnerable period for ASD was associated with pyrethroid exposure of mothers before conception or during the third trimester of pregnancy [18,35,36]. In agreement with these data, previous studies on an animal model exposed to permethrin pyrethroid during early life (from 6th to 21th postnatal day of life, in rats) showed that pesticide, crossing the blood brain barrier, could accumulate in the brain, leading to developmental disorders later in life, characterized by working memory abnormalities [37,38]. Additionally, an intergenerational effect of early life permethrin exposure on offspring was reported, underlining the risk for future generations [39].

Furthermore, toxic metal exposure has been significantly associated with ASD and changes of some microelements likes Mg and Zn, together with toxic metal accumulation (*i.e.*, Al, Cd, and Pb) was observed in hair of autistic children compared to the control healthy group [4,5,6,7,8,9,10,11,12,13]. In the present study, the level of metals and microelements in the hair was studied in another group of ASD and CTR children, living in different Italian areas. The choice to select groups from different areas was intended to avoid any environmental factors that might interfere with the study, and it could be more representative. A significant decrease of Mg in hair of ASD children was found with respect to the controls. These data are in agreement with some reports on ASD children, where the Mg level in hair was significantly reduced in 4–9-years-old children [5,7,8,11]. However this outcome was not in agreement with another study, where authors found insignificant change of Mg in children with ASD 3–15-years-old and 3–6-years-old compared to controls [9].

A larger number of subjects might allow additional information about the other microelements to be gathered, perhaps resolving situations with borderline significance, such as the Ca/Mg ratio that was higher in ASD children (*p* = 0.052), and V, S (*p* = 0.076), and Zn (*p* = 0.09) that were lower in ASD children with respect to controls.

## 5. Conclusions

Our studies underscore that urine and hair represent useful matrices to detect exposure status to xenobiotics of subjects. 

The concentration of the permethrin metabolite 3-PBA in urine was found to be directly correlated with the age in CTR children, while this correlation could not be detected in ASD children, owing to the high variability of data in the ASD population. The increase of 3-PBA with age is likely due to the higher exposure time to this pesticide in their environment or food [40].

Mg was significantly decreased in the hair of ASD children compared to the control group, suggesting that this microelement impairment is associated with ASD.

Further studies involving a larger cohort of subjects will permit to consolidate data on hair concentration of V, S, Zn, and Ca/Mg, that were nearly statistically significant, and to examine the activity of detoxifying enzymes in ASD and CTR children.

## Figures and Tables

**Table 1:** Metals and macro/microelements in hair of ASD and CTR children.

Metals and Macro/Micro-Elements	ASD Mean ± SEM µg/g (ppm)	CTR Mean ± SEM µg/g (ppm)
Calcium (Ca)	409 ± 41.6 °	537 ± 71.0
Magnesium (Mg)	55.3 ± 11.0 *	97.2 ± 18.4
Ca/Mg	9.93 ± 1.02 ^#^	7.86 ± 0.76
Sodium (Na)	n.d.	n.d.
Potassium (K)	n.d.	n.d.
Copper (Cu)	16.9 ± 3.01	25.3 ± 9.85
Zinc (Zn)	103 ± 9.31 ^§^	126 ± 9.57
Iron (Fe)	11.6 ± 1.16	15.2 ± 2.12
Phosphorus (P)	109 ± 6.71	121 ± 5.53
Selenium (Se)	1.41 ± 0.13	1.71 ± 0.21
Manganese (Mn)	0.19 ± 0.03	0.25 ± 0.03
Chromium (Cr)	n.d.	n.d.
Cobalt (Co)	n.d.	n.d.
Molybdenum (Mo)	0.28 ± 0.03	0.29 ± 0.01
Germanium (Ge)	n.d.	n.d.
Sulfur (S)	28614 ± 119 ^$^	31098 ± 783
Vanadium (V)	0.05 ± 0.01 ^$^	0.11 ± 0.03
Barium (Ba)	n.d.	n.d.
Lithium (Li)	0.03 ± 0.01	0.05 ± 0.02
Strontium (Sr)	n.d.	n.d.
Tin (Sn)	n.d.	n.d.
Titanium (Ti)	n.d.	n.d.
Zirconium (Zr)	n.d.	n.d.

n.d. = no differences, * *p* = 0.0382; ° *p* = 0.052; ^#^ *p* = 0.054; ^$^ *p* = 0.076; and ^§^ *p* = 0.09 *vs.* CTR.

**Table 2:** Toxic metals in hair of ASD and CTR children.

Toxic Metals	ASD Mean ± SEM µg/g (ppm)	CTR Mean ± SEM µg/g (ppm)
Mercury (Hg)	0.55 ± 0.13	0.74 ± 0.27
Cadmium (Cd)	0.02 ± 0.01	0.04 ± 0.01
Lead (Pb)	1.09 ± 0.17	1.05 ± 0.14
Beryllium (Be)	n.d.	n.d.
Aluminum (Al)	9.33 ± 1.33	10.4 ± 1.42
Arsenic (As)	0.62 ± 0.16	0.79 ± 0.21
Uranium (U)	0.51 ± 0.09	0.55 ± 0.09
Palladium (Pd)	n.d.	n.d.
Rhodium (Rh)	n.d.	n.d.
Gadolinium (Gd)	n.d.	n.d.
Antimony (Sb)	0.11 ± 0.01	0.13 ± 0.02
Gold (Au)	n.d.	n.d.
Nickel (Ni)	0.29 ± 0.05	0.27 ± 0.04
Platinum (Pt)	n.d.	n.d.
Silver (Ag)	n.d.	n.d.
Tungsten (W)	n.d.	n.d.

n.d. = no differences.

## Supplementary Materials

The following are available online at www.mdpi.com/1660-4601/13/4/388/s1, Figure S1. Two different SOM grids. On the left the rectangular, or in this case a squared grid, while on the right the hexagonal grid. Figure S2. SOMs referred to the variables Mg (**A**); Cu(**B**); Zn(**C**); Hg (**D**); Pb (**E**); Fe (**F**); Se (**G**); Cd (**H**) and Al (**I**). In the SOMs is possible to distinguish the samples (labelled in red) belonging to two different groups: CTR and AUT (ASD). Moreover two nodes, the number 36 and the number 15, are marked respectively with a white and a yellow squares. The marked nodes even if are the same in all the maps are not in the same position. This fact is based on the “Toroidal” boundary conditions: nodes located on the opposite parts of the grid are neighbors so it is possible to translate nodes vertically and horizontally without changing the relations of neighborhood. Figure S3. The weight vectors for two nodes of the SOM. Node 15 (**A**) has a low value for Mg on the contrary in node 36 (**B**) the weight for Mg is high. The SOM mapping algorithm put in node 36 a sample coming from the CTR group and in node 15 a sample coming from the ASD one. In this representation each bar provides the value of the weight of the indicated element for the neuron reported on the top of the histogram.

## Abbreviations

The following abbreviations are used in this manuscript:

ASD	Autism Spectrum Disorder
GC-MD	Gas chromatography-coupled trap mass detector
ICP-OES	Optical Emission Spectrometry
3-PBA	3-phenoxybenzoic acid
CTR	Control
PCA	Principal component analysis
