# Supplementary material for: Pyrethroid Pesticide Metabolite in Urine and Microelements in Hair of Children Affected by Autism Spectrum Disorders: A Preliminary Investigation
Source: Int J Environ Res Public Health. 2016 Mar 30;13(4):388. doi: 10.3390/ijerph13040388 (PMC4847050; doi:10.3390/ijerph13040388)
Supplement: Supplementary file 1 [file ijerph-13-00388-s001.pdf]

Int. J. Environ. Res. Public Health **2016**, *13*, 388; doi:10.3390/ijerph13040388

Supplementary Materials: Pyrethroid Pesticide Metabolite in Urine and Microelements in Hair of Children Affected by Autism Spectrum Disorders: A Preliminary Investigation

Valentina F. Domingues, Cinzia Nasuti, Marco Piangerelli, Luísa Correia-Sá, Alessandro Ghezzo, Marina Marini, Provvidenza M. Abruzzo, Paola Visconti, Marcello Giustozzi, Gerardo Rossi and Rosita Gabbianelli

"The Self-Organizing Map (SOM), commonly also known as Kohonen network [1,2] is a computational method for the visualization and analysis of high-dimensional data, especially experimentally acquired information." Roughly speaking, SOMs projects high-dimensional data into a two-dimensional map. This procedure is called "mapping" and it preserves the topology of the data in a way that similar data items, or samples, will be mapped to nearby locations on the SOM [3].

The SOM is usually characterized by being a lattice, or grid, consisting of a grid of N^2 nodes (also called neurons or cells) where N is the number of neurons for each side of the lattice. Nodes can be represented as rectangles or hexagons. Each grid node is connected to adjacent grid nodes according to a spatial neighborhood relation: a rectangular node has four neighbors while and hexagonal cell has six neighbors (Figure S1).

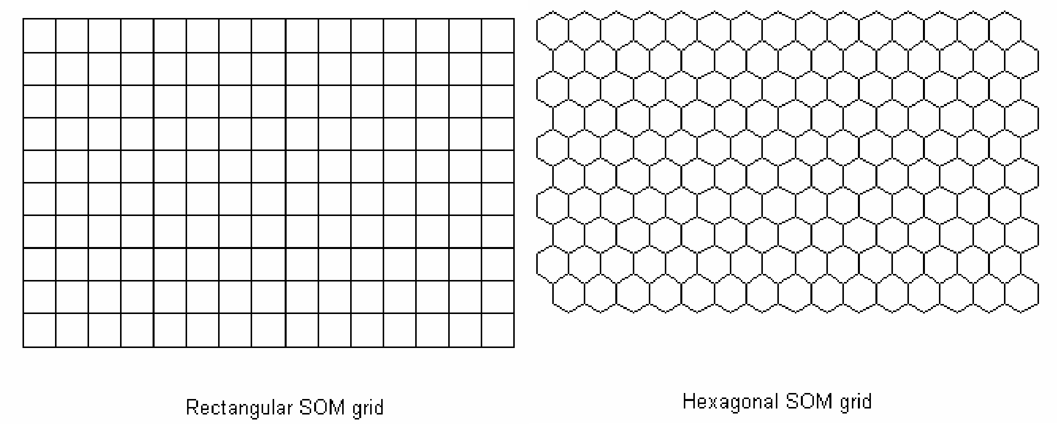

Figure S1. Two different SOM grids. On the left the rectangular, or in this case a squared grid, while on the right the hexagonal grid.

Finally, in describing SOMs, the notion of grid topology has to be taken into account: it is the way we handle the grid boundaries (this concept is very important because, as we will see later on, it has effects on the computation and on the results). Standard topologies supported are:

1. planar: 4 boundaries corresponding to the 4 edges;
2. cylinder: grid with two opposite edges attached, it is like moving on a cylinder;
3. toroidal: grid with both pairs of opposite edges attached, it is like moving on a donut.

1. How do the SOMs Work?

Let $x_i = 100$, with $i = 1,2,\ldots,100$, be the number of samples and the weight, the height, the age, the circumference of the chest and of the pelvis be the measured quantities for each of them. In this way we have 5 variables describing our dataset (it is like having a 100 × 5 matrix). Now, let's consider that we want to do an explorative analysis of our dataset for looking for some possible clusters but without any dimensionality reduction (as in PCA). The use of SOM, allows us, to find clusters hidden in our data and to visualize the contribution of each of the variables in the formation of clusters.

Each node of the SOM contains an adaptive parameter vector whose components have the same cardinality of the variables that we measured in our experiment (according to the example we have a vector with 5 components in each node, one component for each variable). The components of the vectors are also known as the weights of the map and for computation they are initialized randomly between 0 and 1. The mapping procedure consists in presenting all the samples, one at a time, to the SOM for a chosen number of time (the training epochs). For each sample (x_i) the most similar node (*i.e.* the winning node) is selected on the basis of the Euclidean distance (between the sample x_i and the nodes); then, the weights of the r-th neuron (w_r) are changed as a function of the difference between their values and the values of the sample; this correction (Δw_r) is scaled according to the topological distance from the winning neuron (d_r):

$$\Delta w_r = \eta \left(1 - \frac{d_r}{d_{max} + 1}\right)(x_i - w_r^{old})$$

where η is the "learning rate" and d_{max} is the size of the considered neighborhood. The topological distance d_r is defined as the number of neurons between the considered neuron r and the winning neuron. The learning rate η changes during the training phase, as follows:

$$\eta = (\eta^{start} - \eta^{final})\left(1 - \frac{1}{t_{tot}}\right)\eta^{final}$$

where t is the number of the current training epoch, t_{tot} is the total number of training epochs, η^{start} and η^{final} are the learning rate at the beginning and at the end of the training, respectively. At the end of the network training, samples are placed in the most similar neurons of the SOM where similar samples occupy the same nodes or adjacent ones and the weights in each node represents the effect that each variables has on the node. For visualizing the results we need to represent as many SOMs as the number of variables. In all the SOMs the positions of the samples, i.e. the clusters, are the same; the only difference is given by the color scale: it is a way to visualize the influence of the considered variable in the process of clusterization.

2. Results

The SOM algorithm was applied to the present work for clustering the samples into two separate classes on the basis of the experimentally measured variables. SOM analysis was performed with the Matlab Kohonen and CP-ANN Toolbox [4,5], freely available via the internet site [6]. A hexagonal lattice with "Toroidal" boundary condition was employed. The map was trained using the sequential training method and the weights of the network were randomly initialized.

The number of samples is given by the number of people (ASD and CTR) and the number of variables is 9 because we have tested 9 metals (Mg, Cu, Al, Zn, *etc.*). The results of the SOM algorithm was shown in Figure S2. As mentioned above, there are 9 SOMs (one for each measured metal); in almost each node there are some samples (in red) which are mapped by the algorithm in those positions. In reading the results we have to take into account the mapping procedure, *i.e.*, the clusterization, and the influence of the variables on the mapping procedure. The clusterization can be derived by focusing on the red labels, which are arranged in the same way in all the SOMs even if, due to the "Toroidal" boundary conditions, the layout of the maps in Figure S2 seems to be different; the influence of the variables on the clusterization is given by the grey scale: for the each variable darker is the node greater is the influence of that variable. For example looking at the SOM for Mg (Figure S2A) it is reasonable to say that the weight of Mg, higher on the darker nodes, discriminates the samples according to their class: a high number of CTR samples is in the upper left part of the map and belongs to the darker cells while a high number of ASD belongs to the lighter ones and it is located in the lower part right nodes.

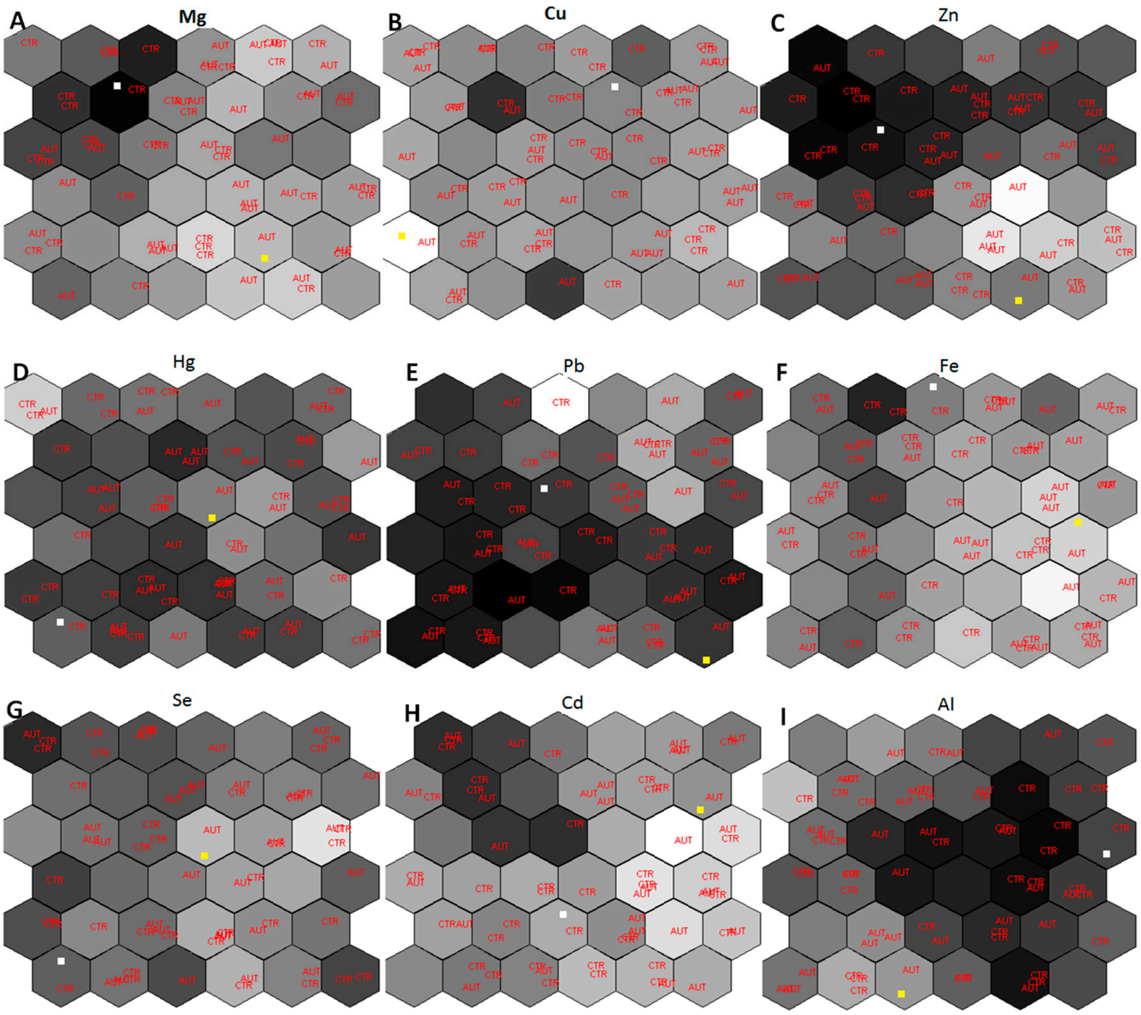

Figure S2. SOMs referred to the variables Mg (**A**); Cu(**B**); Zn(**C**); Hg (**D**); Pb (**E**); Fe (**F**); Se (**G**); Cd (**H**) and Al (**I**). In the SOMs is possible to distinguish the samples (labelled in red) belonging to two different groups: CTR and AUT (ASD). Moreover two nodes, the number 36 and the number 15, are marked respectively with a white and a yellow squares. The marked nodes even if are the same in all the maps are not in the same position. This fact is based on the "Toroidal" boundary conditions: nodes located on the opposite parts of the grid are neighbors so it is possible to translate nodes vertically and horizontally without changing the relations of neighborhood.

Finally, the weight in each node can be represented as histograms. Referring to the marked neurons, in Figure S3 it is possible to observe the distribution of the weights of each variable in the two nodes.

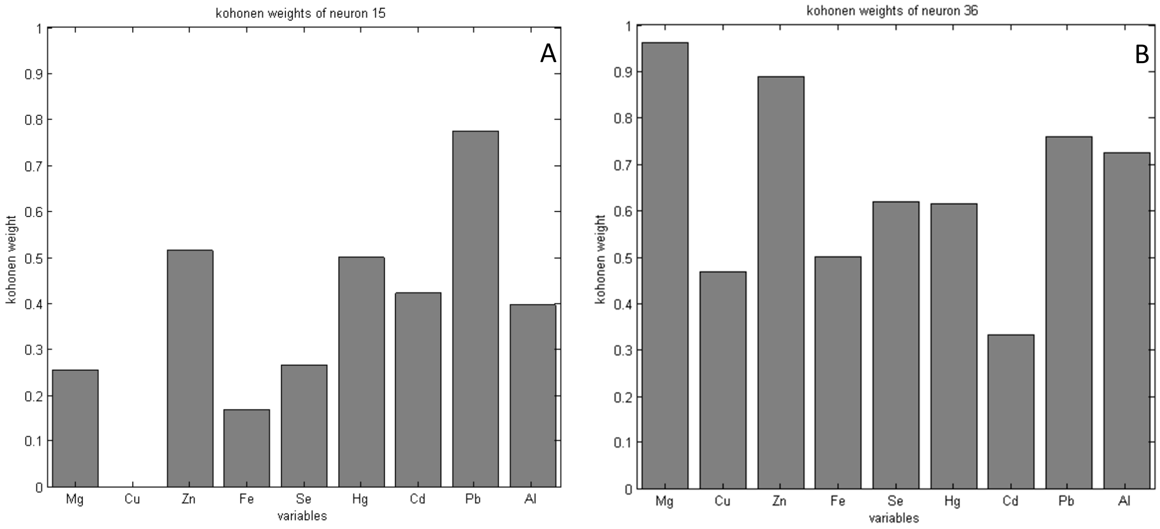

Figure S3. The weight vectors for two nodes of the SOM. Node 15 (**A**) has a low value for Mg on the contrary in node 36 (**B**) the weight for Mg is high. The SOM mapping algorithm put in node 36 a sample coming from the CTR group and in node 15 a sample coming from the ASD one. In this representation each bar provides the value of the weight of the indicated element for the neuron reported on the top of the histogram.

References

1. Kohonen, T. Self-organized formation of topologically correct feature maps. *Biol.Cybern.* **1982**, *43*, 59–69.
2. Kohonen, T. *Self-Organizing Maps*, 3rd ed.; Springer: New York, NJ, USA, 2001.
3. Kohonen, T. *Self-Organization and Associative Memory*; Springer Verlag: Berlin, Germany, 1988.
4. Ballabio, D.; Consonni, V.; Todeschini, R. The Kohonen and CP-ANN toolbox: A collection of MATLAB modules for Self Organizing Maps and Counterpropagation. *Chemometr. Intell. Lab. Syst.* **2009**, *98*, 115–122.
5. Ballabio. D.; Vasighi, M.A. MATLAB Toolbox for Self Organizing Maps and supervised neural network learning strategies. *Chemometr. Intell. Lab. Syst.* **2012**, *118*, 24–32.
6. Milano Chemometrics and QSAR Research Group. http://michem.disat.unimib.it/chm/download/kohoneninfo.htm (accessed on 28 March 2016).

© 2016 by the authors; licensee MDPI, Basel, Switzerland. This article is an open access article distributed under the terms and conditions of the Creative Commons by Attribution (CC-BY) license (http://creativecommons.org/licenses/by/4.0/).
